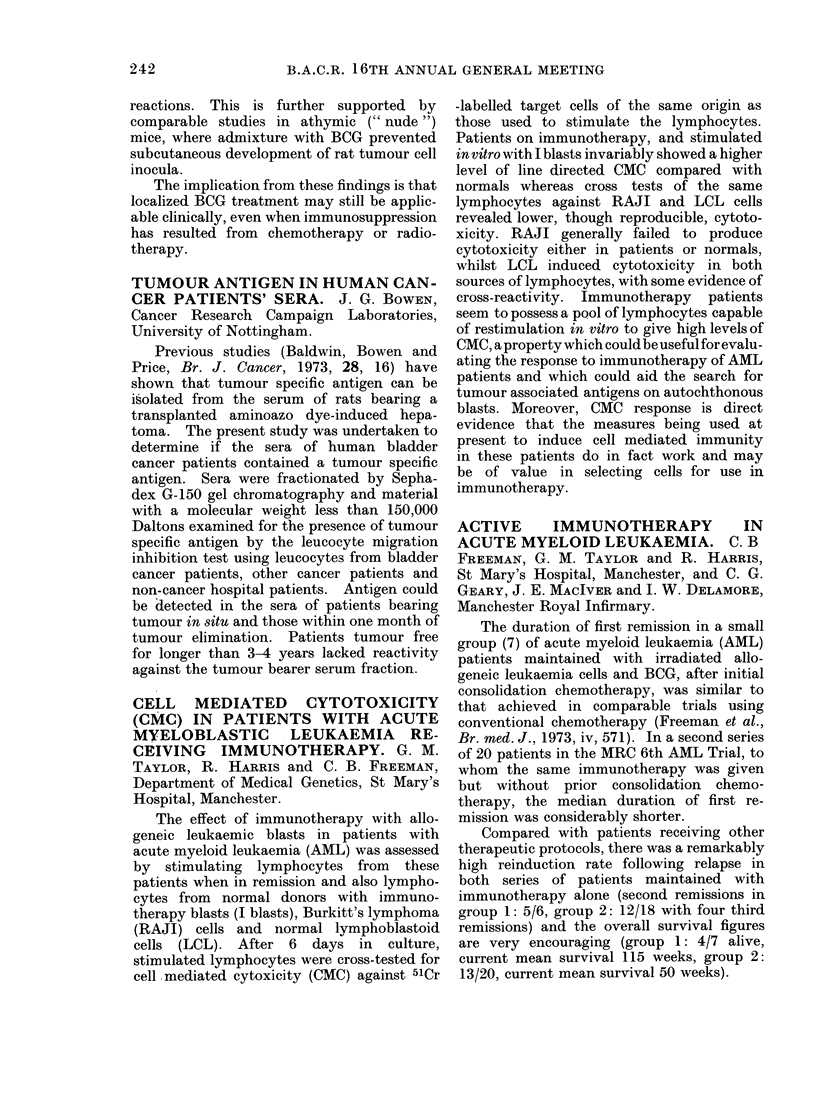# Proceedings: Tumour antigen in human cancer patients' sera.

**DOI:** 10.1038/bjc.1975.161

**Published:** 1975-08

**Authors:** J. G. Bowen


**TUMOUR ANTIGEN IN HUMAN CANCER PATIENTS' SERA.** J. G. Bowen, Cancer Research Campaign Laboratories, University of Nottingham.

Previous studies (Baldwin, Bowen and Price, *Br. J. Cancer*, 1973, **28**, 16) have shown that tumour specific antigen can be isolated from the serum of rats bearing a transplanted aminoazo dye-induced hepatoma. The present study was undertaken to determine if the sera of human bladder cancer patients contained a tumour specific antigen. Sera were fractionated by Sephadex G-150 gel chromatography and material with a molecular weight less than 150,000 Daltons examined for the presence of tumour specific antigen by the leucocyte migration inhibition test using leucocytes from bladder cancer patients, other cancer patients and non-cancer hospital patients. Antigen could be detected in the sera of patients bearing tumour *in situ* and those within one month of tumour elimination. Patients tumour free for longer than 3–4 years lacked reactivity against the tumour bearer serum fraction.